# Building capacity for health equity analysis in the WHO South-East Asia Region

**DOI:** 10.4103/2224-3151.255342

**Published:** 2019-04-01

**Authors:** Devaki Nambiar, Ruchita Rajbhandary, Theadora Swift Koller, Ahmad Reza Hosseinpoor

**Affiliations:** 1 George Institute for Global Health, New Delhi, India; 2 Independent consultant, Kathmandu, Nepal; 3 Gender, Equity and Human Rights, World Health Organization, Geneva, Switzerland; 4 Division of Data, Analytics and Delivery, World Health Organization, Geneva, Switzerland

**Keywords:** equity, health equity assessment, health inequalities, South-East Asia

## Abstract

“Leaving no one behind” is at the heart of the agenda of the Sustainable Development Goals, requiring that health systems be vigilant to how interventions can be accessed equitably by all, including population subgroups that face exclusion. In the World Health Organization (WHO) South-East Asia Region, inequalities can be found across and within countries but there has been a growing commitment to examining and starting to tackle them. Over the past decade in particular, WHO has been developing an armamentarium of tools to enable analysis of health inequalities and action on health equity. Tools include the Health Equity Assessment Toolkit in built-in database and upload database editions, as well as the Innov8 tool for reorientation of national health programmes. Countries across the region have engaged meaningfully in the development and application of these tools, in many cases aligning them with, or including them as part of, ongoing efforts to examine inequities in population subgroups domestically. This paper reflects on these experiences in Bangladesh, India, Indonesia, Nepal, Sri Lanka and Thailand, where efforts have ranged from workshops to programme reorientation; the creation of assemblies and conferences; and collation of evidence through collaborative research, reviews/synthesis and conferences. This promising start must be maintained and expanded, with greater emphasis on building capacity for interpretation and use of evidence on inequalities in policy-making. This may be further enhanced by the use of innovative mixed methodologies and interdisciplinary approaches to refine and contextualize evidence, with a concomitant shift in attention, developing solutions to redress inequities and anchor health reform within communities. There are many lessons to be learnt in this region, as well as mounting political and popular will for change.

## The case for looking at equity

Equity is at the heart of the agenda of the 2030 Sustainable Development Goals (SDGs), to “leave no one behind”, and encompassed in a number of goals and targets related to poverty, health, education, gender, equality and partnerships (SDGs 1, 3, 4, 5, 10 and 17).^1–4^ Progressive realization of the SDGs – especially universal health coverage, as part of the health goal (SDG 3) – requires that health systems be continuously vigilant towards intervention coverage and outcomes for the population overall, and also that they specifically look at whether the situation is improving for population subgroups.^5^

This is especially important in light of what has been known for a long time about the relationship between equity and health reform. Victora and colleagues postulated the “inverse equity hypothesis” many years ago, whereby health reform interventions that are not expressly designed with an equity orientation “will initially reach those of higher socioeconomic status and only later affect the poor. This results in an early increase in inequity ratios for coverage, morbidity, and mortality indicators, followed later by a reduction when the poor gain greater access to the interventions and the rich reach minimum achievable levels for morbidity and mortality, beyond which there are unlikely to be substantial further improvements.”^6^

As various interventions are rolled out in service of the goals of universal health coverage, therefore, the inverse equity hypothesis is something that countries must assess in relation to various socioeconomic groups, but possibly in relation to other population subgroups, identified by sex, age, education and place of residence.^7^ Further, given the SDG goal to “leave no one behind”, it is essential to go beyond the common dimensions of inequality and look specifically from a country and regional context. Some dimensions of inequality are more context specific, such as migrant status, race, ethnicity, caste, religion or other characteristics that can differentiate minority subgroups.

**Fig. 1:**
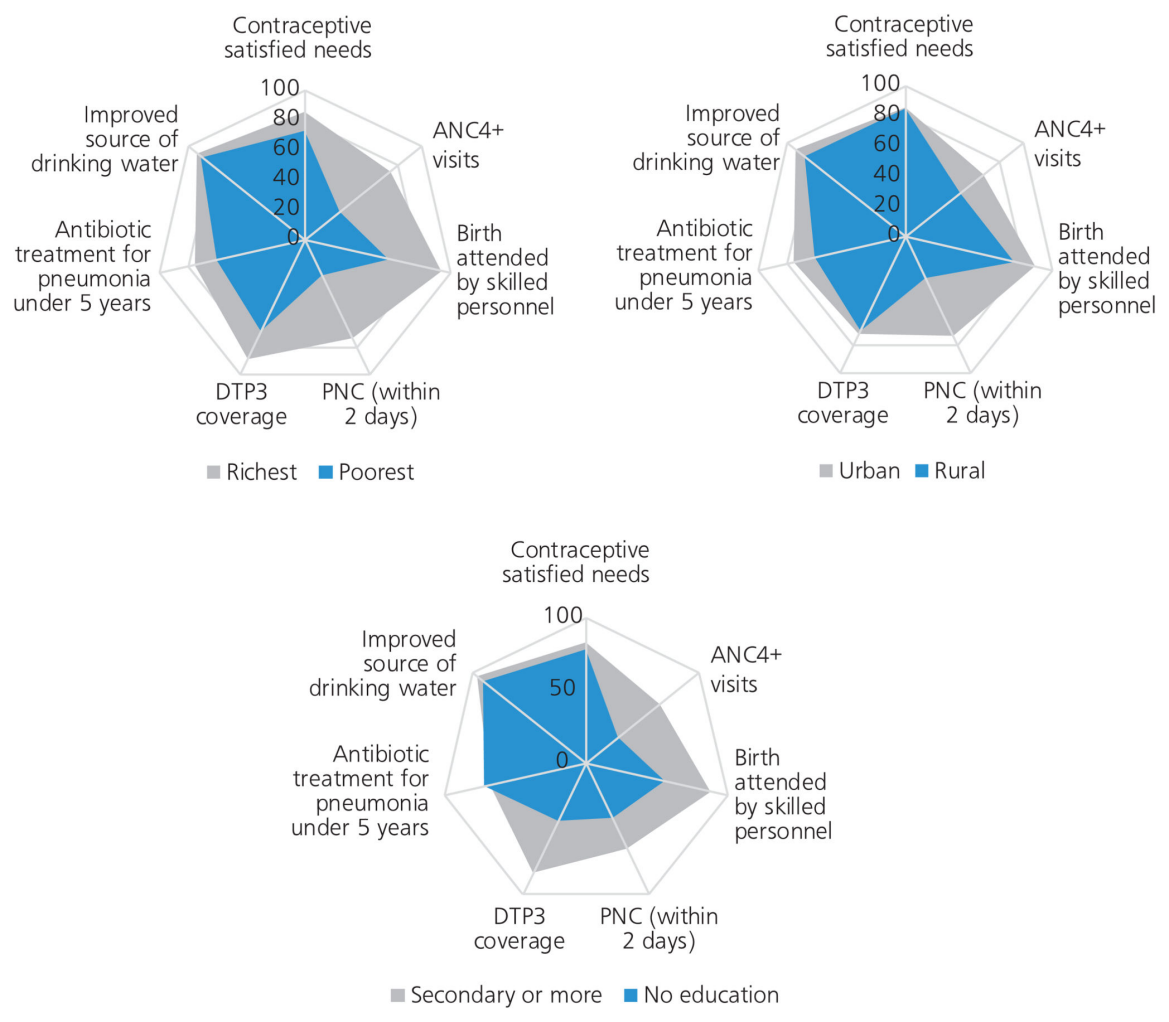
Inequalities in coverage of seven essential areas of health service coverage by income group, urban versus rural location and level of education, across the WHO South-East Asia Region ANC: four or more antenatal care visits; DTP3: diphtheria–tetanus–pertussis vaccination; PNC: postnatal care *Source*: Monitoring progress on universal health coverage and the health-related Sustainable Development Goals in the South-East Asia Region: 2018 update. New Delhi: World Health Organization Regional Office for South-East Asia; 2018 (http://apps.who.int/iris/bitstream/handle/10665/274313/9789290226628-eng.pdf?sequence=6&isAllowed=y).^8^

Health-related inequalities exist within all countries, as well as across countries at varying degrees. Fig. 1 illustrates seven areas of health service coverage in the World Health Organization (WHO) South-East Asia Region, comparing three dimensions of inequality: income group, place of residence (urban/rural) and level of education. Understanding these differences, and how they vary over time and among geographic areas when benchmarked against each other, is the first step in acting with an equity orientation.

## Tools for equity analysis: contributions from WHO

While WHO has been supporting Member States with health inequality monitoring for many years, the use and development of various tools to support this and linked processes has been given greater emphasis over the past 5 years. Across WHO regions, capacity-building workshops have been held; a package of resources, including a handbook, a step-by-step manual and an e-learning module, has been created; and numerous tools to facilitate analysis and visualization of health inequalities have been released.^9–13^ Countries of the WHO South-East Asia Region have played a pivotal role in shaping the nature of capacity-building on health inequality monitoring and the tools that have been created for this purpose.^12,14–17^

A key milestone in this process has been development of the Health Equity Assessment Toolkit (HEAT), a free and open-source software package, updated in 2018, that displays disaggregated data and summary measures of inequality by six dimensions (economic status, education, place of residence and subnational region, as well as age and sex [where applicable]).^12^ HEAT draws from the WHO Health Equity Monitor database,^18^ which includes disaggregated health data based on re-analysis of over 330 demographic and health surveys, multiple indicator cluster surveys and reproductive health surveys carried out between 1991 and 2015, with as many as nine rounds of data for Bangladesh. In 2017, HEAT Plus was launched; this edition of the software allows bespoke data for any indicator (health or otherwise) to be uploaded into a simple template and used for analysis of disaggregated data and summary measures.^11^ HEAT Plus was developed based on extensive inputs from the WHO South-East Asia Region.^14^ Another long-standing effort has been the Innov8 package for review and reorientation of national health programmes aiming to “leave no one behind”.^19^ Innov8 involves an eight-step process where baseline data on a programme are gathered, its theory of change is understood, and then redesign of this theory is collaboratively developed using an equity-oriented, rights-based, gender-responsive approach, mindful of the social determinants of health and measures to monitor, evaluate and ensure sustained attention to leaving no one behind.^19^ The Innov8 approach has been used to increase equity orientation of policies in a number of countries globally.^19–21^

## Experiences of countries in the WHO South-East Asia Region

There has been a series of capacity-building events in the region related to health equity, supported by WHO. Table 1 illustrates the capacity-building activities carried out using WHO tools in the region. While efforts are under way throughout the region, this paper focuses on a few illustrative country examples with which the authors have greater familiarity and indicates the lessons they offer.

### Bangladesh

The Government of Bangladesh has demonstrated its commitment to achieving universal health coverage by 2032.^23^ One of the three main objectives set is to improve equity. In 2017, WHO Bangladesh conducted a workshop on monitoring health inequalities. Prior efforts to look at health equity were already under way in the country, including a Health Equity Watch for which a bespoke survey was carried out in 2002.^24^ Following this, in 2006, the Bangladesh Health Watch (BHW) was launched to monitor health systems and equity-linked reform in the country. To date, BHW has produced analytical reports on health equity (2006); the health workforce (2007); governance of the health sector (2009); universal health coverage (2011); and urban health (2014). The most recent report in 2016 focuses on challenges raised by noncommunicable diseases for service provision at the primary health-care level; regulatory frameworks; and establishing a comprehensive and integrated surveillance system.^25^ Future steps in the country will seek to synergize these efforts and create momentum across government, academia and civil society for equity-oriented change in the country.

### India

In India, an important stream of work on health equity began with a Health Equity Watch workshop held in collaboration with the WHO country office in Delhi in 2013, followed by regional capacity-building on monitoring health inequality. Following this, extensive case-study documentation of research and action on health equity and the social determinants of health was carried out, culminating in a series of publications,^26–29^ and the eventual formation, in 2018, of Health Equity Network India (HENI).^30^ Key findings from these publications include the greater emphasis on socioeconomic inequality in current Indian research; the overall disconnect between research and policy-making; and the fact that many models of concerted action on social determinants of health do exist, but are not identified as such, not evaluated and not considered at scale. Various studies with hard-to-reach populations are under way. A programme of work to carry out health inequality monitoring at the district and subdistrict level in the state of Kerala, as part of monitoring comprehensive primary health-care reforms, was also initiated early in 2018.^31^ At the national level, the newly launched India Strategy for Women’s Adolescents’ and Children’s Health places emphasis on disaggregated data and has a separate set of recommendations on ensuring inclusion.^32^

### Indonesia

In Indonesia, there was enthusiastic adoption of health inequality monitoring, culminating in the creation of a *State of health inequality* report in 2017,^33^ followed by a special issue of the journal *Global Heath Action* showcasing the various analyses undertaken as part of this exercise.^14–17,34^ In addition to HEAT Plus,^11^ the Innov8 tool^19^ was also used for analysis and reorientation of the country’s action plans for neonatal and maternal health and the *National Action Plan on School Aged Children and Adolescent Health 2017–2019*,^35^ to “leave no one behind”.^11,21^ Processes have revealed key geographic areas of the country that require additional attention, as well as emerging areas of emphasis (water and sanitation, adolescent smoking) where inequalities exist.^15,17^ Finally, a conversation around health inequalities has allowed more granular analysis of the Public Health and Development Index in the state, which was designed for programmatic priority-setting and can now be looked at in light of not just average index scores, but also inequalities in the score across regions.^16^ Currently, the government is looking at how to develop subnational analyses using the HEAT Plus toolkit and various other data sources that are relevant for more local decision-making.

### Nepal

Through the Nepal Health Sector Strategy, the Government of Nepal has made a commitment to universal health coverage ensuring health for all.^36^ As Nepal is transitioning towards federalism, it provides an opportunity to restructure and centre the health systems around the principles of universal health coverage. In response to this, the WHO Nepal country office conducted a two-day workshop in 2018 to build local government staff capacity on health inequality monitoring and equity analysis, using HEAT Plus.^11^ This built on a pilot study in 2015 of Nepal’s adolescent sexual and reproductive health programme using the WHO Innov8 tool,^19^ which allowed identification of target subpopulations of adolescents who were either being missed by the national programme, or received suboptimal benefit from the programme.^20^ These recommendations were incorporated in the revised Adolescent Development and Health Strategy in 2017.^37^ Similarly, in 2016, the government established the Nepal Non-communicable Disease and Injuries Poverty Commission, which, in its 2018 report, identified 23 potential cost-effective interventions to be introduced and/or incrementally intensified by 2030, with an emphasis on the poor.^38^

### Sri Lanka

In Sri Lanka, there was interest around the upload database edition of the toolkit – HEAT Plus^11^ – and the possibility of using this for facility-based data that exist in the country. At the capacity-building event hosted by this Member State in December 2017, the health department was able to assemble a broad range of stakeholders, including individuals from the statistics, excise and police departments, demonstrating emerging demand for consideration of health equity across sectors. At the workshop, emphasis was placed on gains made in reducing wealth-related inequalities in satisfaction with modern methods of family planning between 2007 and 2016, while also noting growing inequalities across districts. For other indicators, like stunting among children aged under 5 years, district-level inequalities were highlighted and emphasis placed on regions of the country where tea estates dominated: it appeared that these communities needed greater emphasis in service coverage. Beyond family planning and child health, participants also discussed intimate partner violence, where it was observed that the poorest quintile was more likely than all other wealth groups to report violence in 2016. At this stage, it is unclear whether this is related to instrumentation (comprehension of the question), or operationalization of the indicator (which referred to never experiencing violence from an intimate partner), or truly reflects a phenomenon of concern. Whatever the case, it is clearly a key area for further study and policy action.

### Thailand

Thailand has served as a frame of reference for many others seeking to bring greater equity considerations into routine programming, particularly in relation to universal health coverage. For example, the country’s model of national health assemblies represents a key framework within which public engagement with decision-making and priority-setting in health can take place.^39^ Academics from this country have served as trainers for regional workshops on health inequality monitoring. Further, the Prince Mahidol conference for various years has been related to equity – in the context of neglected diseases, underserved populations, transformative learning, the legacy of comprehensive primary health care, political economy/health-in-all/whole-of-society approaches, and more.^40^ The 2017 conference also featured training on health inequality monitoring. Thailand also uses several data sources to monitor three dimensions of universal health coverage: population coverage, service coverage and financial risk protection.^41^ There are many lessons for process and country stewardship here, not just for Thailand, but also for the region.

## Moving the equity agenda forward

Rising inequities across the world have become a defining challenge of our time. They are putting sustainable development at risk, stirring social unrest, undermining social progress, threatening economic and political stability, and undercutting human rights.^4^ While evidence shows that income inequality *between* countries has been falling in recent decades, income inequality *within* countries (across income levels) has been rising, reaching unprecedented levels in the post-World War II period. A significant majority of households in developing countries – more than 75% of the population – are living today in societies where income is more unequally distributed than it was in the 1990s.^42,43^ Hence, in South-East Asia, improving capacity for equity analysis for health, by income and other intersecting stratifiers, is timely and needed as reforms are undertaken towards universal health coverage.

As these country experiences have revealed, there is great need, moving forward, to carry out more finely grained analyses of equity as an integral part of universal health coverage-relevant policy implementation and decision-making at national and subnational levels. Here, there is great value in indicator-based quantitative monitoring, as well as qualitative forms of monitoring, such as social audits, community-based monitoring and mixed methods barrier assessments. The use of qualitative evidence of strong quality can also help to move beyond discussions around magnitudes of inequalities to mechanisms, contexts, structures and strictures that allow inequities to both proliferate and perpetuate. In the WHO South-East Asia Region, there is growing recognition of this and demand for interdisciplinary approaches to understand and act on health inequities.

This being the case, as one member of the Health Equity Network India put it, “pathways to health inequities are different from pathways to health equity”. This is an important notion, not just in conceptual, but also in practical terms. Health inequality monitoring is but a first step in determining the root causes of inequalities that can point towards, but are inadequate to fully determine, how equity may be promoted. Sustained and community-led research and programmatic activity on promoting health equity is an essential step in moving forward and must include the development and testing of approaches and processes that foster distribution of resources according to need and are accountable to outcomes at the population level. Equity must remain centre-stage – not just rhetorically, but de facto – in the emerging agenda of universal health coverage, anchored in the legacy of Alma-Ata^44^ and the continuing movement towards health for all. There are many lessons to be learnt in this region, as well as mounting political and popular will for change.

## Figures and Tables

**Table 1:** Timeline of capacity-building and development of tools for health inequality monitoring and programme reorientation in the WHO South-East Asia Region

			Scope		
Dates	Activity	Location/scope	National	Regional	Global
April 2013	Health equity workshop	India	×		
April 2014	Health inequality monitoring workshop	India		×	
November 2014 to 2017	Development of Innov8 tool^19^	Various		×	
January to July 2015	Development of HEAT^12^ (Beta)	WHO headquarters			×
June 2015	HEAT^12^ launch	United States of America			×
September to November 2015	Innov8^19^ analysis	Nepal	×		
June 2015 to March 2016	Development of HEAT Plus^11^ (Beta)	Indonesia	×		×
April 2016	HEAT (Plus)^11^ workshop	Indonesia	×		
June 2016	Innov8^19^ and AA-HA! programme^22^ to reach every adolescent	India		×	
December 2016	Innov8^19^ launch	WHO headquarters			×
January 2017	Health inequality monitoring workshop	Thailand		×	×
February 2017	HEAT^12^ and HEAT Plus^11^ workshop	India		×	
July 2017	HEAT Plus launch	WHO headquarters			×
August 2017	HEAT Plus^11^ workshop	Bangladesh	×		
December 2017	HEAT Plus^11^ workshop	Sri Lanka (with a delegation from Myanmar)	×	×	
January 2018 – ongoing	Subnational application of HEAT Plus^11^	India	×		
September 2018	HEAT Plus^11^ workshop	Nepal	×		
October 2018	Subnational application of HEAT Plus^11^	Indonesia	×		

AA-HA!: Global Accelerated Action for the Health or Adolescents;^22^ HEAT: Health Equity Assessment Toolkit;^12^ Innov8: tool for reviewing national health programmes to leave no one behind.^19^
